# Anatomical and Biomechanical Characteristics of Peroneus Longus Tendon: Applications in Knee Cruciate Ligament Reconstruction Surgery

**DOI:** 10.1155/2023/2018363

**Published:** 2023-06-27

**Authors:** Quan Nguyen Hoang, Khanh Nguyen Manh

**Affiliations:** ^1^Hanoi Medical University, Hanoi 100000, Vietnam; ^2^Department of Upper Limb Surgery and Sports Medicine, Viet Duc University Hospital, Hanoi 100000, Vietnam

## Abstract

**Introduction:**

The peroneus longus tendon is used in many orthopedic surgeries to regenerate the external ligaments of the knee. This study aims to evaluate some anatomical, biomechanical, and load-bearing properties of the peroneus longus tendon for use in cruciate ligament reconstruction.

**Materials and Methods:**

The study design is a cross-sectional description. The study subjects were 20 samples of the peroneus longus tendon from fresh carcasses. The leg is still intact, not crushed, is well preserved, and it has never been used in research.

**Results:**

The average length of the peroneus longus tendon was 29.25 ± 2.1 cm, and the average distance from the peroneus longus tendon to the deep peroneal nerve was 71.1 ± 8.63 mm. The peroneus longus tendon did not have an accessory ligament, the maximum tension of the peroneus longus tendon was 1170.4 ± 203 N, and the maximum length at break was 14.29 ± 3.88 mm.

**Conclusion:**

Removing the peroneus longus tendon will not affect the surrounding anatomical components. The maximum breaking force and the diameter of the peroneus longus tendon are similar to other graft materials, such as the hamstring tendon and patellar tendon.

## 1. Introduction

Knee injuries account for 41% of sports injuries, of which anterior cruciate ligament injuries account for 20–50% [1, 2]. Today, arthroscopic reconstruction of the knee cruciate ligament has been widely applied, and advances in arthroscopic surgery have brought many good results to patients. However, the choice of grafting material is still controversial.

Autograft tissues commonly mentioned in studies include the bone-patellar tendon-bone graft, the hamstrings, and the quadriceps tendon [3], and current graft materials are not considered ideal materials to replace the cruciate ligament. These sources of tendon graft alone are not enough when both anterior and posterior cruciate ligaments must be reconstructed simultaneously or in case of revision anterior cruciate ligament surgeries. Besides, although the hamstring is the most commonly used grafting material because of its similar characteristics and suitable mechanical strength, this material still has some disadvantages related to the flexibility of the knee joint [4, 5], injury to the sartorial (terminal) branch of the saphenous nerve [6], slower soft-tissue graft-tunnel healing, and increased laxity over time [7]. Patellar ligament graft was first used in 1963 by Jones; since then, this material has been widely used in ligament reconstruction surgery. Besides the advantages of strength, stiffness, and potential for bone integration [8], this material also has disadvantages, such as patellar fractures, weakening of the quadriceps muscles, patellar tendon rupture, and patellar tendonitis [9, 10]. Several studies suggest that the quadriceps tendon may represent a versatile alternative graft in primary and revision anterior and posterior cruciate ligament reconstruction [11, 12]. However, surgeons often choose something other than this material for several reasons. First, the harvesting technique is complex, leading to a longer operative time [13]. The second drawback is the need for trials with long-term follow-up to demonstrate efficacy and show complications associated with tendon extraction [14, 15].

Recently, the peroneus longus tendon (PLT) is also considered an appropriate graft option. PLT grafts in knee ligament surgery are becoming increasingly popular, with studies showing tensile strength [16] and favorable functional outcomes [17–20]. However, most studies have not adequately evaluated evidence on the anatomical and biomechanical features of the PLT that are essential parameters for surgical performance. This study aims to describe the anatomical, biomechanical, and load-bearing properties of the PLT to be applied to knee cruciate ligament reconstruction.

## 2. Materials and Methods

### 2.1. Study Settings

The study design is a cross-sectional description. The subjects of the study were 20 limb specimens from the Department of Anatomy, University of Medicine and Pharmacy, Ho Chi Minh city. The fresh carcasses were selected at random, and the selection criteria were fresh carcasses and adult amputated limbs above the knee, without injury to the lateral aspect of the lower leg. Exclusion criteria were fresh carcasses and amputations that have been dissected or poorly preserved. The study was approved by the Ethics Committee of Hanoi Medical University under decision no. NCS09/BB-HDĐ.

### 2.2. Study Procedure and Data Collection

Surgical instruments include surgical knife, Metzenbaum scissors, Kelly forceps, surgical forceps with nodules, dissecting instruments, needle-bearing forceps, mechanical dynamometer, length measuring cloth (mm), drawing pen, lead color, ribbing tools, and measuring tendons.

We performed a dissection on the outside of the leg to record anatomical parameters. First, we determined the anatomical landmarks, the lateral malleolus top and the fibula head, and then made a skin incision to expose the outer surface of the lower leg from the lateral malleolus top to the fibula head, exposing the superficial peroneal nerve and the calf nerve, lateral peroneal muscle, and deep peroneal nerve. Through the deep fascia, we can see the PLT. We cut across the PLT at the apex of the lateral malleolus; then, we used a tendon extractor to extract the tendon and continued dissection to locate the tip of the tendon, the deep peroneal nerve.

### 2.3. Evaluation Criteria

- Additional grip bands of the PLT
- Length and diameter of the PLT in normal and 4-folded state
- Distance from the apex of the tendon extractor to the deep peroneal nerve
- Degree of damage to tendons and adjacent structures after tendon extraction
- The load-bearing force of the PLT in the quadruple flexion state, measured by a testometric machine (the tendon is fixed to the two ends of the traction machine by homemade tools, the PLT will be lengthened at both ends at the same time until the machine shows signs of tendon rupture, and the force measured at that time is calculated as the load-bearing force of the PLT)

### 2.4. Statistical Analysis

The results were coded and processed by STATA software. Quantitative variables are described as the mean and standard deviation if the results are normally distributed or as the median if not. Qualitative variables are described by frequency and percentage.

## 3. Results

Among the 20 limb specimens, the mean age was 38.9 ± 11.2 and the male/female ratio was approximately 1 : 1.

**Figure 1:**
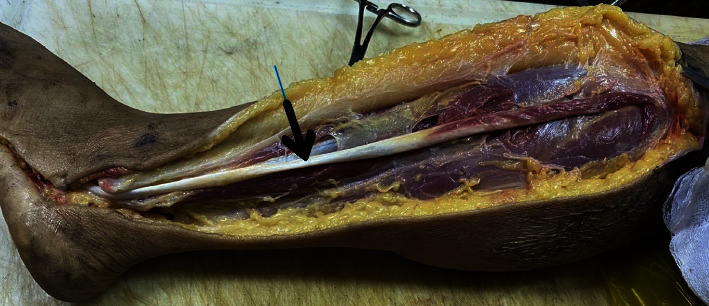
Peroneus longus tendon (arrow) with no appendages above the lateral malleolus.

The peroneus longus muscle originates from the head of the fibula, the end point is the cuneiform bones, and the base of the first metatarsal and the lower part (from about the middle 1/3) gradually turns into a tendon; from above the lateral malleolus, it is all tendon. The PLT extends from the head of the fibula to the back of the lateral malleolus, and there is no accessory attachment to the entire tendon of the lower leg (Figure 1).

**Figure 2:**
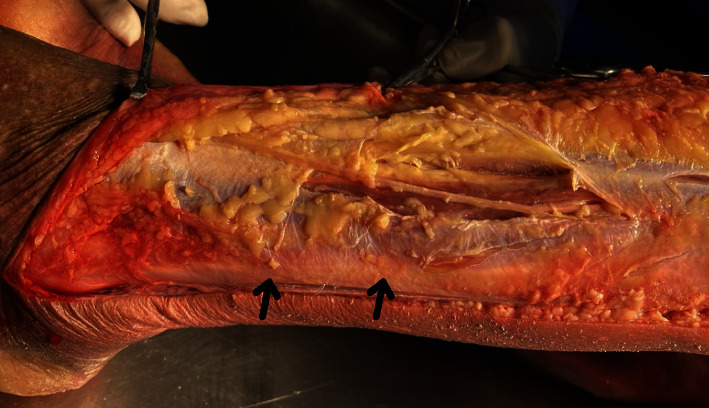
Anatomy of the peroneus longus tendon (arrow).

Short Fibula Muscle. In the superior lateral and posterior lateral malleolus, the PLT is shallower than the peroneus brevis tendon. The fascia that holds the peroneal tendon is located behind the lateral malleolus (Figure 2).

**Figure 3:**
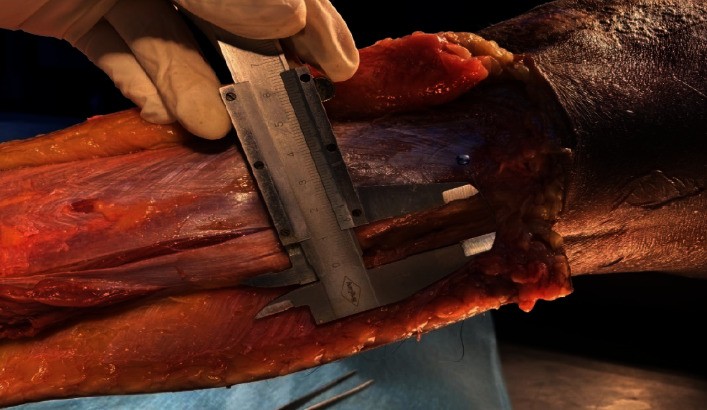
Distal lateral calf cutaneous nerve at the ankle.

External Calf Nerve. At 1.5 cm superior to the lateral malleolus, the PLT is located at about 20 ± 2 mm from the calf dermal nerve (Table 1), and at 12 cm superior to the lateral malleolus, the PLT is located distant from the calf dermal nerve at about 40 ± 2 mm (Figure 3).

**Figure 4:**
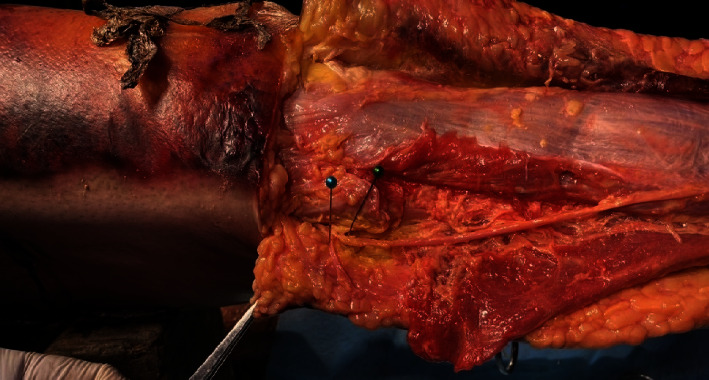
Common peroneal nerve.

**Figure 5:**
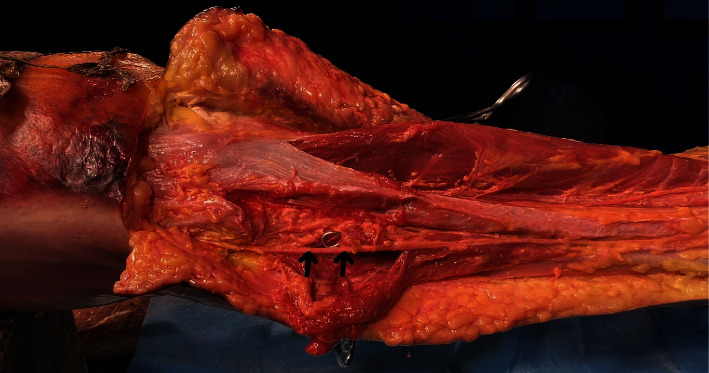
Superficial peroneal nerve (arrow) passes between the long and short peroneal muscles.

Thus, in the direction from the lateral malleolus towards the fibula head, the calf dermal nerve is gradually further away from the PLT; so, it is unlikely that this nerve will be injured during the process of taking the tendon. The common peroneal nerve, after circumventing the fibula head, divides into the superficial peroneal nerve and the deep peroneal nerve. The superficial peroneal nerve passes between the long and short peroneal muscles and gives a branch that innervates this muscle, descending inferiorly and anteriorly, through the superficial fascia. The nerve is not completely transversed directly to the tendon of the long peroneal muscle. The deep peroneal nerve separates from the common peroneal nerve approximately perpendicular to the course of the PLT (Figure 4). Therefore, if the tendon extractor is pointed too far, there is still a risk of injury to this nerve branch (Figure 5), and the average distance from the PLT to the deep peroneal nerve is 71.1 ± 8.63 mm (Table 1) with a minimum distance of 48 mm and a maximum of 86 mm.

The length of the PLT was taken for the study from the tip of the fibula to the point where there was no muscle on the tendon surface, just above the lateral malleolus (Table 1).

The PLT, when folded in four, has an average diameter of 9.95 ± 1.27 (Table 1), the smallest being 8 mm and the largest being 13 mm. If only 2/3 of the PLT is taken, the average diameter measured is 7.83 ± 0.77 with the largest diameter measured being 9.5 mm (Table 2).

## 4. Discussion

All 20 tendon samples obtained were of standard quality, the tendons were completely normal, the tendon quality was good, and there was no phenomenon of tendon stripping. After taking the PLT, we performed further dissection to identify damage to the calf dermal nerve, superficial peroneal nerve, deep peroneal nerve, and blood vessels or adjacent structures. The results showed no damage to nerves or blood vessels around.

The results from the study showed that the average length of the PLT was 29.25 ± 2.1 cm; if folded in half, the size is about 14-15 cm, and if folded in four, the length will be approximately 7-8 cm. In the study of Zhao and Huangfu, the author used the anterior half of the PLT as the autologous graft source. The length of the anterior half of the PLT was 23.7 ± 1.4 cm [20]. Goyal et al. conducted a study using PLT grafts to reconstruct the anterior cruciate ligament, and the average length of the PLT obtained in the study was 26.2 cm (ranging from 22 to 31) [17].

In terms of shape, the muscle attaches at the top and the upper third of the fibula, and the lower part gradually turns into a tendon. With the path going from behind the lateral malleolus to the tip of the fibula, the middle 1/3 of the muscle gradually turns into a tendon, and from the position above the lateral malleolus, it is all tendons. Based on this feature, when taking tendons, we direct the tendon extractor from the lateral malleolus to the tip of the fibula. From the lateral malleolus to the tip of the fibula, the PLT has no accessory attachments, which is an advantage over the hamstrings because the hamstrings have many attachments, and this can make it difficult to remove the tendon as well as reduce the quality of the tendon after extraction. The study of Zhao and Huangfu also noted that the PLT did not have any secondary attachment [20]. The mean distance from the tendon to the deep peroneal nerve was 71.1 ± 8.63 mm, the shortest being 48 mm and the farthest 86 mm. This distance is relatively safe if we do not accidentally push the tendon extractor too far. In fact, when performing surgery, we only make a small incision of about 2 cm, which is enough to remove the tendon, and a large dissection to observe and avoid damage to other structures is not necessary. Goyal et al. in the study using the PLT for anterior cruciate reconstruction also used only a skin incision of about 3 cm behind the lateral malleolus [17]. The study of Zhao and Huangfu also confirmed that the removal of the PLT did not affect the surrounding nerves [20].

One of the most important factors that need considerations during ligament reconstruction is the diameter of grafts. Many studies have shown that the diameter of the graft is related to the outcome of surgery [21–23]. In this study, we found that when taking the entire PLT in fours, the diameter of the tendon is quite large (9.95 ± 1.27 mm). In some cases, the tendon diameter is too large and will affect the results of cruciate ligament reconstruction surgery because when the intercondylar notch is too small, it may cause the tendon to be crushed after reconstruction or cause pain for the patient. Besides, removing the entire PLT may affect the function of the ankle [24]. To solve this problem, the research team only took the anterior 2/3 of the PLT for grafting because taking the anterior 2/3 ensured the required length and did not lose the raise function of the foot. The anterior 2/3 of the PLT when sutured in quadruples will have an average diameter of 7.83 ± 0.77 (minimum 6.5 mm and maximum 9.5 mm) with an average length of about 60–70 mm suitable for the minimum length required for all-insides ligament reconstruction. The results of measuring the diameter of the PLT between studies varied depending on the graft preparation technique. Bi et al. measured a mean diameter of 7.9 mm [24]. In a study by Song et al. on 156 patients who underwent anterior cruciate ligament reconstruction surgery with a 4th fibula tendon graft, the results showed that the average diameter of the graft was 8.3 mm, of which 13.5% of the patients had a diameter of less than 8 mm; 54.5% of the patients had a diameter between 8 and 9 mm, and 32.0% of the patients had a diameter greater than or equal to 9 mm [25].

Another factor that is also very important when choosing a grafting material is its tensile strength. Previous studies have shown that the process of graft incorporation reduces the biomechanical properties of the graft [26, 27]. In our study, the maximum load-bearing force of the PLT was 1170.4 ± 203 N, and the tendon length, when subjected to full tension, was 14.29 ± 3.88 cm (Table 3). Pearsall et al. [28] studied three types of allograft tendons used in knee surgery, including the anterior tibial tendon in two folds, the posterior tibial tendon in two folds, and the PLT in two folds. The study sample consisted of 16 fresh frozen carcass legs and 16 tendon fragments in two folds of the corresponding limb. The results show that these grafts have a greater maximum bearing capacity than the anterior cruciate ligament. In the study of Zhao and Huangfu [20], the load-bearing force of 1/2 PLT was 322.35 ± 63.18 N. In a study by the author Oliver Morgan, the mean failure load of the PLT was 723 N [29]. This difference is related to the tendon fixation method, race, and age group. Rudy et al. in 2017 conducted a study comparing the tensile strength between the PLT and the hamstring tendon, using six human cadaveric specimens as the research material. The authors took the hamstring and the PLT from both lower extremities and then used the Hydraulic Servo Pulser tensile test apparatus to measure the tensile strength. The results show that the maximum load capacity of the PLT is 446.16 ± 233.28, while the maximum load capacity of the hamstring is 405.88 ± 202.92 [16]; the difference is not statistically significant with *p*=0.656. The load-bearing force of the PLT in this study was much lower than in our study because the authors measured on a single fiber tendon, while in our study, the PLT was folded in 4 (the quadrupled peroneus longus tendon). Phatama et al. in 2019 compared the tensile strength between four types of hamstring, patellar tendon, quadriceps tendon, and PLT. 48 tendon samples were obtained from 6 cadavers (12 pieces for the quadriceps tendon, 12 for the hamstring, 12 for the PLT, and 12 for the patellar tendon). The results showed that the tensile strength of the PLT was not significantly different from that of the hamstring tendon but was considerably higher when compared with the patellar and quadriceps tendon [30].

Our study also has some limitations, such as a small sample size, lack of comparison between the states of the same tendon or different types of tendons, and the correlation between the characteristics of tendons and clinical features such as age and sex has not been analyzed. Further studies in the future are needed to supplement the data mentioned previously, providing a complete view of the PLT's characteristics and helping clinicians choose a better graft material when performing ligament reconstruction techniques.

## 5. Conclusion

The PLT graft can be used as a material in knee cruciate ligament reconstruction to replace the traditional hamstring tendon. Anatomically, the PLT does not have ancillary fissures from the lateral malleolus up to the fibula head, and the extent of tendon extraction does not affect the surrounding superficial and deep peroneal nerves. The breaking force and length at the maximal stretch of the PLT are both suitable as grafts in knee cruciate ligament reconstruction.

## Figures and Tables

**Table 1:** Dimensions of the peroneus longus tendon.

Peroneus longus tendon	Mean	Min	Max
Length (cm)	29.25 ± 2.1	26	33
Distance to the deep peroneal nerve (mm)	71.1 ± 8.63	48	86
Distance to the calf dermal nerve (mm)	20 ± 2	17	22

**Table 2:** Dimensions of the peroneus longus tendon when folded in 4.

Peroneus longus tendon	Diameter (mm)	
	Mean	(Min–max)
Entire tendon folded in 4	9.95 ± 1.27	(8.0–13.0)
2/3 tendon folded in 4	7.83 ± 0.77	(6.5–9.5)

**Table 3:** Maximum length at break and breaking force.

2/3 peroneus longus tendon folded in 4		Min–max
Length (mm)	14.29 ± 3.88	8.01–21.37
Breaking force (N)	1170.4 ± 203	1006.8–1720

## Data Availability

The datasets used and/or analyzed during the current study are available from the corresponding author upon reasonable request.

## Abbreviations

PLT: Peroneus longus tendon.
